# Retraction: Ultrasound-Facilitated Catheter-Directed Thrombolysis via Dual Right Upper Extremity Venous Access Into the Basilic Vein in a Case of Submassive Pulmonary Embolism

**DOI:** 10.7759/cureus.r23

**Published:** 2021-03-05

**Authors:** Ahmed Elkhouly, Derek Enos, Justin Fox

**Affiliations:** 1 Internal Medicine, St. Francis Medical Center, Trenton, USA; 2 Cardiology, St. Francis Medical Center, Trenton, USA

This article has been retracted based on the discovery that the submitting author, Dr. Ahmed Elkhouly, invited his wife to serve as a peer reviewer without properly disclosing this relationship. As this fraudulent peer review was completed and taken into consideration when determining whether to publish this article, Cureus has no choice but to retract this article due to this author misconduct and falsification of peer review.

An additional four articles submitted by Dr. Elkhouly have been retracted for the same reason. Cureus greatly regrets that these fraudulent peer reviews were not identified prior to publication. Dr. Elkhouly's residency program has been notified as is consistent with COPE guidelines.

